# Adolescent Isolation Interacts With *DISC1* Point Mutation to Impair Adult Social Memory and Synaptic Functions in the Hippocampus

**DOI:** 10.3389/fncel.2018.00238

**Published:** 2018-08-02

**Authors:** Nan Li, Lin Cui, Ge Song, Li Guo, Huating Gu, Haisheng Cao, Guo-Dong Li, Yu Zhou

**Affiliations:** ^1^Department of Physiology and Pathophysiology, School of Basic Medical Sciences, Qingdao University, Qingdao, China; ^2^Department of Pathology, Qingdao Municipal Hospital, Affiliated to Medical College of Qingdao University, Qingdao, China; ^3^Department of Surgery, Valley Presbyterian Hospital, Van Nuys, CA, United States; ^4^Institute of Brain Sciences and Related Disorders, Qingdao University, Qingdao, China

**Keywords:** *DISC1*, L100P, social interaction, social memory, adolescent stress

## Abstract

Disrupted-in-schizophrenia 1 (*DISC1*) is a strong candidate susceptibility gene for a spectrum of neuropsychiatric diseases including schizophrenia, bipolar disorder and major depression, all of which are thought to result from interactions between gene mutations and environmental risk factors such as influenza, trauma and stress. Adolescence is a key period susceptible to stress and stress-related mental illnesses. In a previous study, we found that although *DISC1* L100P point mutation mice shows object recognition deficits, their sociability and social memory are relatively normal. Therefore, in this article, we investigated whether the interaction between adolescent stress and *DISC1* L100P point mutation affects adult social memory, and we explored the underlying mechanisms. We found that adolescent stress (isolation from 5 weeks to 8 weeks of age) specifically impaired social memory of adult *DISC1* L100P mice but not that of WT littermates, which could be rescued by administration of atypical antipsychotic drug clozapine. On the other hand, it did not induce anxiety or depression in adult mice. Adolescent isolation exacerbated adult neurogenesis deficits in the hippocampus of *DISC1* L100P mice, while it had no effect on WT mice. In addition, we found that adolescent isolation led to long lasting changes in synaptic transmission and plasticity in the hippocampal circuits, some of which are specific for *DISC1* L100P mice. In summary, we identified here the specific interaction between genetic mutation (*DISC1* L100P) and adolescence social stress that damages synaptic function and social memory in adult hippocampal circuits.

**Highlights**

– Adolescent isolation (from 5 weeks to 8 weeks of age) impairs adult social memory when combined with *DISC1* L100P point mutation.
– Adolescent isolation exacerbates adult neurogenesis deficit in the hippocampus of L100P mice but has no similar effect on WT mice.
– Adolescent isolation causes long lasting changes in synaptic transmission and plasticity of the hippocampal network in *DISC1* L100P mice.

## Introduction

Major neuropsychiatric illnesses such as schizophrenia are genetically complex but they share not only overlapping symptoms and environmental risk factors, but also molecular etiology (Tropea et al., 2016). Among many genes susceptible to these disorders (Gogos and Gerber, 2006; Haque et al., 2012), Disrupted-in-schizophrenia 1 (*DISC1*) has been identified as one of the most prominent ones according to linkage and association studies in multiple pedigrees (Roberts, 2007; Cash-Padgett and Jaaro-Peled, 2013).* DISC1* was originally discovered in a large Scottish family with identical chromosomal translocation but very different clinical features (St Clair et al., 1990), indicating that gene-environment interactions might be a potential mechanism underlying the complex heritability and variable phenotypes of psychiatric disorders.

Gene × environment studies have been done with both *DISC1* transgenic mice (Abazyan et al., 2010; Ibi et al., 2010; Nagai et al., 2011; Niwa et al., 2013) and *DISC1* point mutation (L100P and Q31L) heterozygotes (Haque et al., 2012; Lipina et al., 2013). Previous studies reported that Q31L homozygous showed depression-like behaviors while L100P homozygous showed schizophrenia-like phenotype (Clapcote et al., 2007). However, subsequent studies from another independent group reported normal behaviors of both Q31L and L100P mutants in general (Shoji et al., 2012), suggesting that the influence of *DISC1* point mutation itself on behaviors is not very robust and it may depend on environmental factors. Supportively, in our previous study, we found that although *DISC1* L100P mice show object recognition deficits, their locomotor activity, spatial learning and memory, sociability and social memory are relatively normal (Cui et al., 2016).

Adolescence is a sensitive neurodevelopment period associated with plasticity-driven organization of neural circuits in multiple brain regions (Pattwell et al., 2011; Selemon, 2013; Kozareva et al., 2017). Besides perinatal immune activation (Abazyan et al., 2010; Ibi et al., 2010; Nagai et al., 2011; Lipina et al., 2013), adverse experience during adolescence also influences postnatal brain maturation and increases risk for stress-related mental illnesses in adulthood (Blakemore, 2008; van Os et al., 2010; Niwa et al., 2013). In particular, social stress during adolescence are central features for depression, anxiety, schizophrenia and addiction (Burke et al., 2017).

A previous study reported that isolation stress during adolescence elicited molecular, neurochemical and behavioral deficits only when combined with *DISC1-DN* mutation (Niwa et al., 2013). It is interesting to test whether the same social stress during adolescence has long lasting effects on both behavior and stress-related neural circuits in *DISC1* L100P mice. Since L100P homozygotes exhibit relatively normal behaviors, we used these mice to explore the synergistic interplay between genetic and environmental risk factors. We hypothesized that *DISC1* point mutation would affect vulnerability to adolescent stress.

## Materials and Methods

### Animals

Male *DISC1* L100P homozygous in C57BL/6J background were obtained from RIKEN BRC^1^ and were backcrossed to inbred C57BL/6J female mice from Jackson Laboratory for one generation. The resultant heterozygous progeny (L100P/+) were intercrossed to generate L100P/L100P, L100P/+ and +/+ littermates. Mice were group-housed after weaning and maintained on a 12 h light/12 h dark cycle with free access to food and water. All animal protocols were approved by the Chancellor’s Animal Research Committee at the university, in accordance with National Institutes of Health guidelines.

### Adolescent Social Isolation

Mice were isolated from 5 weeks to 8 weeks of age for 3 weeks and maintained single-housed afterwards to avoid fighting. Behavioral tests were conducted 4 weeks later when the mice were 12 weeks old (Niwa et al., 2013). We studied four groups: WT mice without social isolation (WT or control); WT mice with isolation (WT-iso, environmental stressor E only); *DISC1* L100P mice without isolation (L100P, genetic factor G only); and *DISC1* L100P mice with isolation (L100P-iso, G × E).

### Behavioral Tests

All behavioral analyses were done with adult male mice. Tests were performed between 9:00 am and 6:00 pm. Animal behaviors were video-tracked and analyzed with Noldus EthoVision XT software.

An elevated plus maze (EPM) test was conducted as described previously (Zhao et al., 2014). Time spent in the open or close arm, number of arm entries, and total travel distance were calculated over 5 min. In open field (OF) tests, total distance traveled, time spend in the center or peripheral area, vertical and stereotyped activity were analyzed over 10 min (Zhao et al., 2014). Forced swimming (FS) test were conducted as previously described (Zhou et al., 2013). Total immobility time and the latency to first immobility were analyzed over 5 min.

Social behaviors were assessed according to previous publication with minor modifications (Arime et al., 2014). The test consisted of three sessions: habituation, sociability and social novelty preference (SNP). In habituation, mice were allowed to freely explore for 5 min. In sociability, mice were introduced to an unfamiliar, ovariectomized female mouse enclosed in the center and allowed to freely explore for 10 min. SNP test was conducted 24 h after sociability. Mice were encountered with two ovariectomized female mice: one had met in sociability test (familiar), the other was a stranger. The locations of the two female mouse were counterbalanced. Social interaction time was analyzed over 10 min.

Novel object recognition (NOR) and object-place recognition (NPR) tests were conducted according to previous studies (Cui et al., 2016). During training, mice were exposed to two identical objects for 10 min. During NOR test 1 h after training, one object was changed to a new one that the animal has never met; while during NPR test 24 h after training, two objects were the same, but one stayed in the same location as during training (old location), while the other one was moved to a new location (new location). Both NOR and NPR test last for 5 min.

### BrdU Injection and Immunofluorescence Staining

5-bromo-2′deoxyuridien (BrdU, 100 mg/kg) was injected for 5 days at both the 8th week and the 11th week of age, twice every day at 8 h intervals. BrdU were freshly dissolved in normal saline (0.9%, pH 7.4).

Hippocampal coronal sections (50 μm) from each brain were collected in sequence and divided into four equivalent sample sets. Each set included 8–10 sections at 200 μm intervals to cover the entire anterior-posterior extent of dentate gyrus (DG; Zhao et al., 2014). Free-floating sections were pre-treated with 1 M HCl (fresh) for 30 min at 45°C. Primary antibodies were rat anti-BrdU monoclonal antibody (1:500; Accurate Chemicals), rabbit anti-doublecortin (anti-DCX) polyclonal antibody (1:500, Abcam) and rabbit anti-NeuN monoclonal antibody (1:1000, Abcam). Secondary antibodies were alexa-488 chicken anti-rat and alexa-568 goat anti-rabbit (1:500, Abcam). Fluorescence images were acquired with a laser confocal microscope (FV500, Olymus) and the associated Fluoview2000 software. The objective lens used was 20× and 40×.

We quantified all BrdU^+^, BrdU^+^/DCX^+^ and BrdU^+^/NeuN^+^ cells throughout the anterior-posterior extent of the granule cell layer according to previous studies (Kee et al., 2007; Zhou et al., 2013). To get the average number of labeled cells per section, we first counted the total number of positive cells from each set of brain sections, and then divided it by the number of sections within that set. Finally, the total number of labeled cells per entire DG was quantified by multiplying this average number of labeled cells per section by 10. We used Z-stack function to scan at least eight focal planes per section, and the three-dimension images were checked carefully to exclude false double labeling. Cell quantification was conducted by two independent investigators unaware of the experimental design.

### Hippocampus Slices Preparation and Electrophysiological Recordings

Hippocampal slice was prepared according to previous description (Zhou et al., 2007; Cui et al., 2016). Briefly, coronal slices (400 μm in thickness) were sectioned with a vibratome (VT-1000, Leica) in an oxygenated ice-cold cutting solution (pH 7.4) containing (in mM) 119 choline chloride, 2.5 KCl, 26 NaHCO_3_, 1 NaH_2_PO_4_, 7 MgSO_4_, 1 CaCl_2_, 30 Glucose, 3 sodium pyruvate, 1 kynurenic acid and 1.3 sodium L-ascorbate. Slices were quickly transferred to a recovery solution containing (in mM) 85 NaCl, 2.5 KCl, 24 NaHCO_3_, 1.25 NaH_2_PO_4_, 4 MgCl_2_, 0.5 CaCl_2_, 25 glucose and 50 sucrose to recover for 30 min at 30°C and then at least 1 h at room temperature prior to recording.

Whole cell patch-clamp recordings were conducted in both CA1 pyramidal neurons and DG granule cells. The glass micropipettes (4–6 MΩ) was filled with an internal solution (pH 7.3) containing (in mM) 130 CsMeSO_4_, 10 CsCl, 4 NaCl, 1 MgCl_2_, 5 MgATP, 5 EGTA, 10 HEPES, 0.5 Na_3_GTP, 10 phosphocreatine and four QX-314. Artificial cerebral spinal fluid (ACSF) consists of (in mM) 120 NaCl, 3.5 KCl, 2.5 CaCl_2_, 1.3 MgSO_4_, 1.25 NaH_2_PO_4_, 26 NaHCO_3_ and 10 glucose. ACSF was perfused with a flow rate of ~2 ml/min at 31–33°C.

sIPSCs were recorded at a holding potential of +20 mV with 3 mM kynuric acid; while sEPSCs were recorded at a holding potential of −70 mV with 50 μM AP-5 and 50 μM picrotoxin. mIPSCs and mEPSCs were recorded with 1 μM TTX. Only recordings with series resistance changes less than 20% throughout the experiment were analyzed using Mini Analysis Program. Event counts were carried out by an experimenter blind to genotypes.

Evoked AMPA and NMDA currents were recorded in the presence of 100 μM picrotoxin. AMPA current was measured as peak amplitude at −70 mV; NMDA current was determined by amplitude at 100 ms after the onset of stimulation at +30 mV. The AMPA/NMDA ratio was calculated by averaging 10–15 events.

The input-output curve in field recordings was constructed by varying stimulus intensity and measuring the initial slopes of fEPSPs. PPR were determined by fEPSP2/fEPSP1. LTP at SC-CA1 synapses was induced by 100 Hz tetanus. All stimuli were 100 μs in duration and 1/3–1/2 of maximal stimulation strength (100 μA). Analog to digital conversion was performed using Digidata 1440A. Data were filtered at 2 kHz and digitized at 10 kHz with Clampex10. The experimenters were blind to genotypes of the mice. All chemicals used in electrophysiological recordings were purchased from Sigma.

### Statistical Analysis

Results are expressed as mean ± SEM. Data was analyzed using one-sample *t*-test, unpaired *t*-test, one-way ANOVA, or two-way ANOVA as appropriate. *P* < 0.05 indicates significant difference. Statistical analysis was performed with Graphpad Prism 7.0.

## Results

### Adolescent Isolation Specifically Impaired Social Memory of *DISC1 L100P* Mice but Had No Effect on WT Littermates

**Figure 1:**
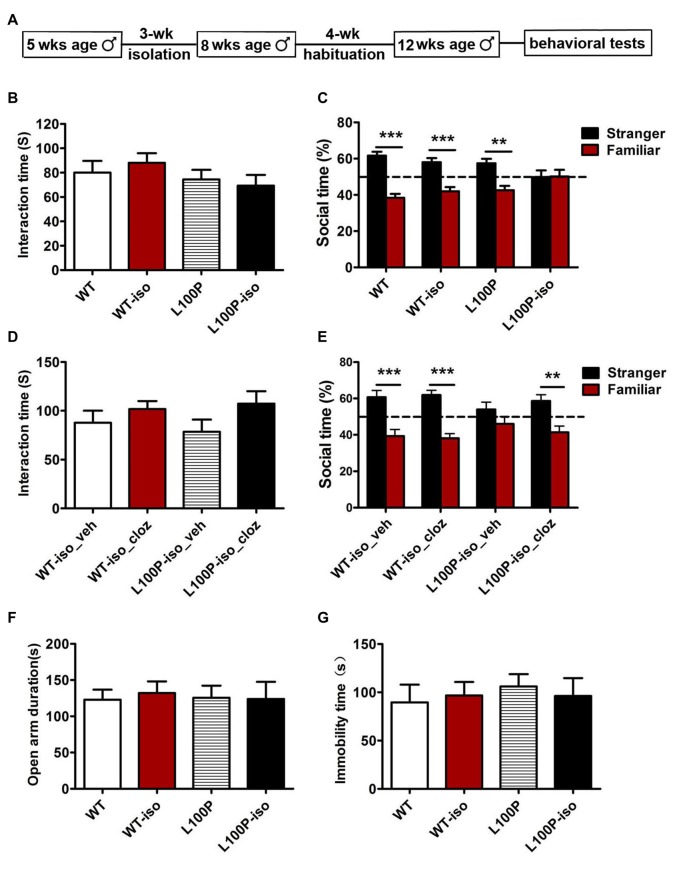
Adolescent isolation specifically impaired social memory in adult L100P mice. **(A)** Behavioral procedures. **(B)** Sociability test. One-way ANOVA, *n* = 8 for each group, *P* > 0.05. **(C)** Social memory test showing L100P-iso mice had no novelty preference. Two-way ANOVA, *n* = 8 for each group, ***P < 0.001 or **P < 0.01. **(B,C)** WT-iso and L100P-iso represent WT and L100P mice exposed to adolescent isolation, WT and L100P represent non-exposure groups. **(D)** Clozapine had no effect on sociability. One-way ANOVA, *P* > 0.05. **(E)** Clozapine rescued social memory deficit in L100P-iso mice. Two-way ANOVA, **P < 0.01 or ***P < 0.001. **(D,E)** WT-iso_veh (*n* = 8), WT-iso_cloz (*n* = 10), L100P-iso_veh (*n* = 8) and L100P-iso_cloz (*n* = 10) represent isolated WT or L100P mice receiving vehicle or clozapine treatment respectively. **(F)** elevated plus maze (EPM) test. **(G)** forced swimming (FS) test. **(F,G)** One-way ANOVA, *P* > 0.05, *n* = 8 for each group. All data are shown as means ± SEM.

A previous study reported that adolescent isolation for 3 weeks caused affective behavioral deficits in adult *DISC1-DN-Tg-PrP* mice that showed otherwise normal behaviors (Niwa et al., 2013). Using their strategy, we single housed *DISC1* L100P male mice and WT littermates from 5 weeks of 8 weeks of age. Behaviors were tested 4 weeks later when mice were 12 weeks old (Figure 1A). We found that, 3-week isolation during adolescence did not affect sociability: all four groups spent similar time interacting with the ovariectomized female mice (Figure 1B; one-way ANOVA, *F*_(3,31)_ = 0.876, *P* > 0.05). However, L100P-iso mice exhibited profound deficits in SNP test: L100P-iso mice interacted with the stranger and the familiar equally (Figure 1C; two-way ANOVA, *F*_(1,56)_ = 49.61, *P* < 0.001; Bonferroni post-tests, *t* = 0.09, *P* > 0.05); while the other three groups all spent significantly more time interacting with the stranger than with the familiar (Figure 1C; *P* < 0.01 to *P* < 0.001). The total interaction time during SNP test were comparable among four groups (data not shown), suggesting that social memory deficit observed in L100P-iso mice were not caused by impaired exploration/sociability due to isolation. Consistent with our previous report (Cui et al., 2016), L100P and WT mice showed similar performance in sociability and social memory tests (Figures 1B,C). Therefore, our results indicated that isolation during adolescence impaired adult social memory when combined with a genetic risk, in this case, *DISC1* L100P point mutation.

### Clozapine Administration Rescued Social Memory Deficit Caused by Adolescent Isolation in L100P Mice

In previous study, we found that administration of clozapine prevented object recognition deficits in *DISC1* L100P mice (Cui et al., 2016). To test whether antipsychotics treatment could rescue the social memory deficit observed in L100P-iso mice, we administrated clozapine (i.p., 0.6 mg/kg dissolved in 10% DMSO, Tocris) or vehicle into adult WT-iso and L100P-iso mice at 40 min before sociability and novelty preference tests. Our results showed that clozapine had no effect on sociability (Figure 1D; one-way ANOVA, *F*_(3,34)_ = 0.21, *P* > 0.05), but rescued the social memory deficit observed in L100P-iso mice. In comparison to the vehicle-treated L100P-iso mice (L100P-iso_veh) who spent similar time on the stranger as on the familiar (Figure 1E; two-way ANOVA, *F*_(1,62)_ = 52.86, *P* < 0.001; Bonferroni post-tests, *t* = 1.48, *P* > 0.05), the clozapine-treated L100P-iso mice (L100P-iso_cloz) spent significantly more time interacting with the stranger (Figure 1E; *t* = 3.85, *P* < 0.01). Clozapine had no effect on WT-iso mice since both WT-iso_veh and WT-iso_cloz groups spent more time interacting with the stranger than with the familiar (Figure 1E; *t* = 4.28–5.32, *P* < 0.001). The total interaction time during novelty preference test were comparable among four groups (data not shown).

### Adolescent Isolation Did Not Increase Anxiety or Depression in *DISC1* L100P Mice

Since anxiety or depression may affect learning and memory performance, and a previous study showed that social isolation did change anxiety- and depression-like behaviors in *DISC1-DN* transgenic mice (Niwa et al., 2013), we then checked whether the same isolation changes anxiety- or depression-like behaviors of *DISC1* L100P mice. EPM test showed that four groups spent similar time in open arms (Figure 1F; one-way ANOVA, *F*_(3,31)_ = 0.27, *P* > 0.05). FS test showed that the total immobility time of four groups were comparable (Figure 1G; one-way ANOVA, *F*_(3,31)_ = 0.35, *P* > 0.05). Those data indicated that adolescent isolation from 5 weeks to 8 weeks of age did not increase anxiety or depression. Therefore, social memory deficit observed in adult L100P-iso mice could not be explained by changes in anxiety or depression. In addition, our finding suggested that social isolation from 5 weeks to 8 weeks of age may not be adverse enough to affect anxiety- or depression- like behaviors even when combined with *DISC1* L100P point mutation.

### Adolescent Isolation Exacerbated Adult Neurogenesis Deficit in L100P Mice but Had No Effect on WT Mice

**Figure 2:**
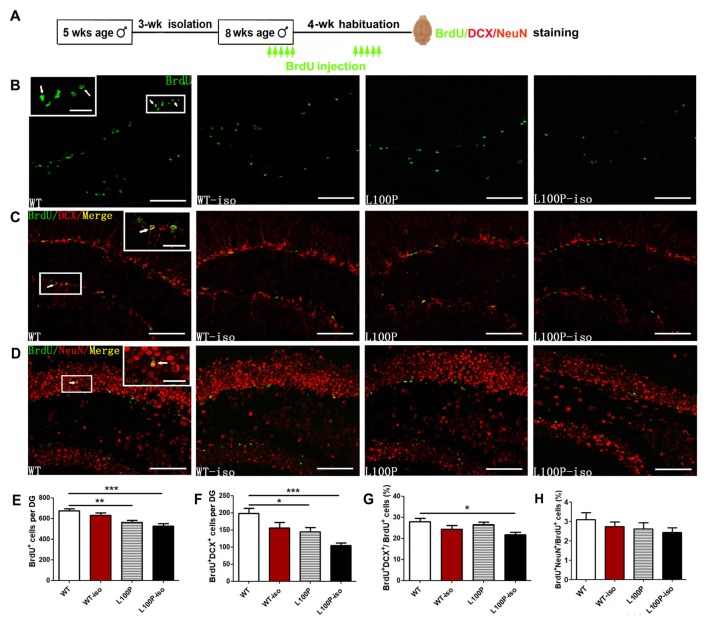
Adolescent isolation exacerbated adult neurogenesis deficit in L100P mice.** (A)** Experimental design. **(B)** 5-bromo-2′deoxyuridien (BrdU^+^ cells immunostaining. **(C)** Immunostainings for BrdU^+^ (green), doublecortin (DCX^+^; red) and BrdU^+^DCX^+^ (yellow) neurons. **(D)** Immunostainings for BrdU^+^ (green), NeuN^+^ (red) and BrdU^+^NeuN^+^ (yellow) neurons. Insets in **(B–D)**, higher magnification of boxed area. White scale bar, 20 μm (inside) and 100 μm (outside). White arrows, the same cell with different magnification. **(E–G)** Reduced number of BrdU^+^ cells **(E)** and BrdU^+^DCX^+^ neurons **(F)**, and reduced BrdU^+^DCX^+^/BrdU^+^ ratio **(G)** in L100P-iso mice. **(H)** BrdU^+^NeuN^+^/BrdU^+^ ratio. **(E–H)** One-way ANOVA, *P < 0.05, **P < 0.01 or ***P < 0.001, *n* = 6 for each group. All data are shown as means ± SEM.

It is well known that stress decreases adult neurogenesis in the hippocampus (Aimone et al., 2014; Kozareva et al., 2018). We also found impaired neurogenesis (reduced BrdU^+^ cells) in adult *DISC1* L100P mice. To test whether social stress during adolescence and *DISC1* L100P mutation has synergistic effect on adult neurogenesis, we did immunostaining and counted newborn cells (BrdU^+^), immature neurons (BrdU^+^DCX^+^) and new neurons (BrdU^+^NeuN^+^) in DG of the hippocampus (Figure 2A). Consistently, adult L100P mice showed less BrdU^+^ cells than WT mice (Figures 2B,E; one-way ANOVA, *F*_(3,20)_ = 10.52, *P* < 0.001; Dunnett’s Multiple Comparison Test, *q* = 3.76, *P* < 0.01). The number of BrdU^+^DCX^+^ neurons were also reduced in L100P mice (Figures 2C,F; one-way ANOVA, *F*_(3,20)_ = 8.95, *P* < 0.001; Dunnett’s Multiple Comparison Test, *q* = 2.94, *P* < 0.05). However, no difference was observed between L100P and WT mice in the percentage of BrdU^+^ cells that adopted a neuronal fate or became a BrdU^+^DCX^+^ double-labeling (Figure 2G; one-way ANOVA, *F*_(3,20)_ = 3.78, *P* < 0.05; Dunnett’s Multiple Comparison Test, *q* = 0.73, *P* > 0.05). All these data suggested that L100P mutation only reduces proliferation or survival of progenitor cells but had no effect on neuronal differentiation. Remarkably, L100P-iso mice showed not only reduced number of BrdU^+^ cells (Figure 2E; *q* = 4.74, *P* < 0.001) or BrdU^+^DCX^+^ neurons (Figure 2F; *q* = 5.15, *P* < 0.001), but also a smaller ratio of BrdU^+^DCX^+^/BrdU^+^ compared to WT (*q* = 3.15, *P* < 0.05). Clearly, social isolation during adolescence did not affect adult neurogenesis in WT mice, the number of BrdU^+^ cells or BrdU^+^DCX^+^ neurons, and the BrdU^+^DCX^+^/BrdU^+^ ratio were all comparable between WT and WT-iso mice (Figures 2E–G; *P* > 0.05). Therefore, our findings suggest that adolescent isolation stress and *DISC1* L100P mutation play a synergistic role to impair both proliferation and neuronal differentiation. We also observed a reduce tendency (not significant yet) in the number of BrdU^+^NeuN^+^ neurons (data not shown) or the ratio of BrdU^+^NeuN^+^ /BrdU^+^ in L100P-iso mice (Figures 2D,H).

### Adolescent Isolation Caused Long Lasting Changes of Synaptic Transmission in Hippocampal Network of L100P Mice

Previously, we have reported abnormal synaptic transmission and plasticity in hippocampal network of L100P mice (Cui et al., 2016). To test whether adolescent isolation and L100P mutation synergistically affect synaptic functions of the adult hippocampus which may underlie social memory deficit observed in L100P-iso mice, we first checked synaptic transmission in four groups of hippocampal slices.

**Figure 3:**
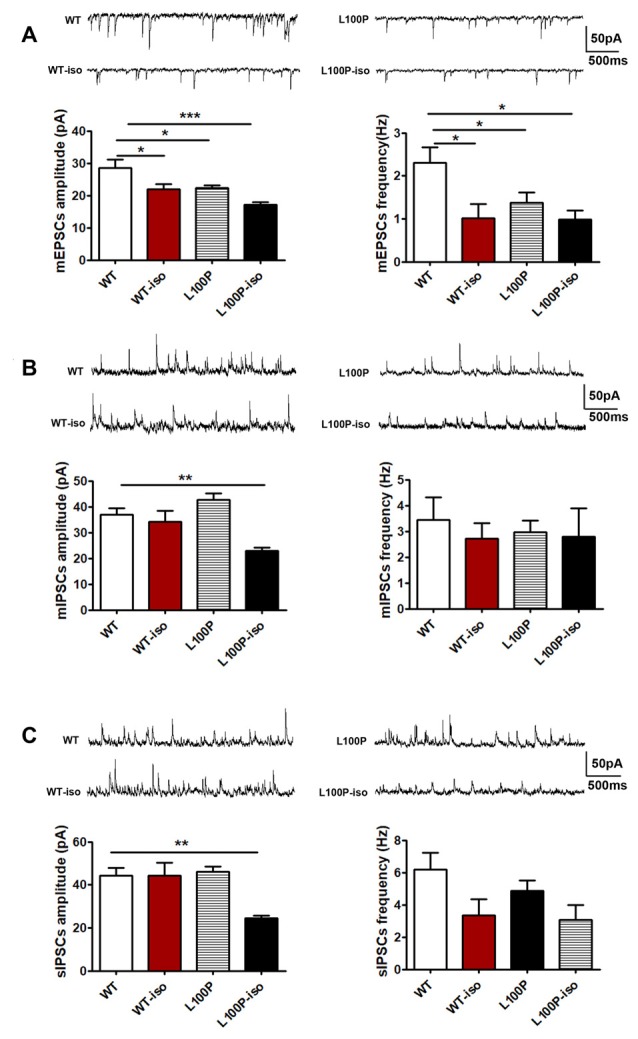
Adolescent isolation caused long lasting changes of synaptic transmission in dentate gyrus (DG) granule cells of L100P mice. **(A)** mEPSCs. Upper: sample traces in four groups. Lower left: mEPSCs amplitude. Lower right: mEPSCs frequency. One-way ANOVA, *P < 0.05 to ***P < 0.001 compared to WT, *n* = 10, 7, 11, 7 slices from five mice per group. **(B)** mIPSCs. Upper: sample traces. Lower left: mIPSCs amplitude. Lower right: mIPSCs frequency. One-way ANOVA, **P < 0.01 compared to WT, *n* = 8, 8, 9, 8 slices from five mice per group. **(C)** sIPSCs. Upper: sample traces. Lower left: sIPSCs amplitude. Lower right: sIPSCs frequency. One-way ANOVA, **P < 0.01 compared to WT. *n* = 11, 8, 9, 8 slices from five mice per group. All data are shown as means ± SEM.

We found that, in DG granule cells, either adolescent isolation (WT-iso), L100P mutation (L100P) or both (L100P-iso) inhibited mEPSCs (both amplitude and frequency) compared to the WT controls (Figure 3A; for mEPSCs amplitude: one-way ANOVA, *F*_(3,31)_ = 8.15, *P* < 0.001; Dunnett’s Multiple Comparison Test, *q* = 2.80–4.87, *P* < 0.05–0.001. For mEPSCs frequency: one-way ANOVA, *F*_(3,31)_ = 4.59, *P* < 0.01; Dunnett’s Multiple Comparison Test, *q* = 2.61–3.10, *P* < 0.05). In contrast, the same isolation suppressed mIPSCs amplitude only when combined with L100P mutation (Figure 3B, one-way ANOVA, *F*_(3,29)_ = 8.55, *P* < 0.001; Dunnett’s Multiple Comparison Test, *q* = 3.40, *P* < 0.01). Neither isolation nor mutation itself affects mIPSCs in granule cells (Figure 3B, *P* > 0.05). Consistently, reduced sIPSCs amplitude were observed in L100P-iso mice (Figure 3C, one-way ANOVA, *F*_(3,32)_ = 7.27, *P* < 0.001; Dunnett’s Multiple Comparison Test, *q* = 3.91, *P* < 0.01).

**Figure 4:**
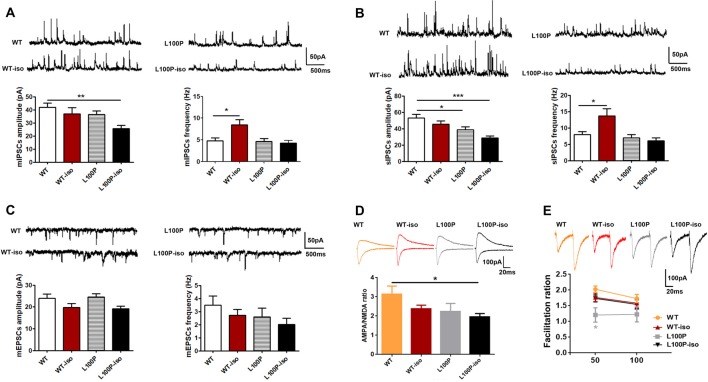
Adolescent isolation caused long lasting changes of synaptic transmission in CA1 pyramidal neurons of L100P mice. **(A)** mIPSCs. Upper: sample traces in four groups. Lower left: mIPSCs amplitude. Lower right: mIPSCs frequency. One-way ANOVA, *P < 0.05, **P < 0.01 compared to WT, *n* = 10, 10, 11, 10 slices from five mice per group. **(B)** sIPSCs. Upper: sample traces. Lower left: sIPSCs amplitude. Lower right: sIPSCs frequency. One-way ANOVA, *P < 0.05 or ***P < 0.001 compared to WT, *n* = 9, 10, 11, 10 slices from five mice per group. **(C)** mEPSCs. Sample traces (upper), mEPSCs amplitude (lower left) and mEPSCs frequency (lower right). One-way ANOVA, *P* > 0.05, *n* = 9, 9, 8, 9 slices from five mice per group. **(D)** The AMPA/NMDA ratio. Upper: sample traces of AMPA and NMDA currents. Lower: reduced AMPA/NMDA ratio in L100P-iso mice. One-way ANOVA, *P < 0.05, *n* = 12, 19, 10, 14 slice from six mice per group. **(E)** PPR. Upper: sample paired-pulse response at an interval of 50 ms. Lower: PPR at intervals of 50 ms and 100 ms. Two-way ANOVA, *P < 0.05 compared to WT, *n* = 11, 25, 8, 18 slices from five mice per group. All data are shown as means ± SEM.

In CA1 pyramidal neurons, we did not find significant difference in mEPSCs among four groups (Figure 4C, *P* > 0.05). Instead, in comparison to WT controls, CA1 pyramidal neurons of L100P-iso mice exhibited reduced amplitude (not frequency) of both mIPSCs (Figure 4A, one-way ANOVA, *F*_(3,37)_ = 4.03, *P* < 0.05; Dunnett’s Multiple Comparison Test, *q* = 3.37, *P* < 0.01) and sIPSCs (Figure 4B, one-way ANOVA, *F*_(3,36)_ = 7.05, *P* < 0.001; Dunnett’s Multiple Comparison Test, *q* = 4.39, *P* < 0.001). Considering that WT-iso neurons showed normal amplitude and increased frequency of both mIPSCs (Figure 4A, one-way ANOVA, *F*_(3,37)_ = 5.38, *P* < 0.01; Dunnett’s Multiple Comparison Test, *q* = 3.02, *P* < 0.05) and sIPSCs (Figure 4B, one-way ANOVA, *F*_(3,36)_ = 6.18, *P* < 0.01; Dunnett’s Multiple Comparison Test, *q* = 2.82, *P* < 0.05), while L100P mutant neurons displayed normal mIPSCs (Figure 4A) and slightly reduced amplitude of sIPSCs (Figure 4B, *q* = 2.66, *P* < 0.05), our data altogether suggested that adolescent isolation causes or worsens abnormal inhibitory synaptic transmission in both DG granule cells and CA1 pyramidal neurons in hippocampal microcircuit, which may contribute to social memory deficit observed in adult L100P-iso mice. Of course, abnormal excitatory synaptic activity in DG granule cells may also underlie social memory deficit in adult L00P-iso mice.

We also found that the AMPA/NMDA ratio was significantly lower in CA1 pyramidal neurons of L100P-iso mice (Figure 4D; one-way ANOVA, *F*_(3,51)_ = 2.92, *P* < 0.05; Dunnett’s Multiple comparison Test, *q* = 2.88, *P* < 0.05), which could reflect a decrease in the AMPA current and/or an increase in the NMDAR current triggered by interaction between adolescent isolation and L100P mutation. In contrast, the AMPA/NMDA ratio was unaltered in WT-iso and L100P mice (Figure 4D, *P* > 0.05).

PPR in CA1 neurons of isolated mice were not changed (Figure 4E; *P* > 0.05 compared to WT), indicating that presynaptic excitatory neurotransmitter release probability was not affected by social isolation. In contrast, PPR in CA1 neurons of L100P mice showed small but significant decrease at inter-pulse interval of 50 ms (Figure 4E; two-way ANOVA, *F*_(3,116)_ = 3.514, *P* < 0.05; Sidak’s multiple comparisons test, *t* = 2.80, *P* < 0.05), which is consistent with our previous finding (Cui et al., 2016).

### Adolescent Isolation Caused Long Lasting Changes of Synaptic Plasticity in Hippocampal Network

**Figure 5:**
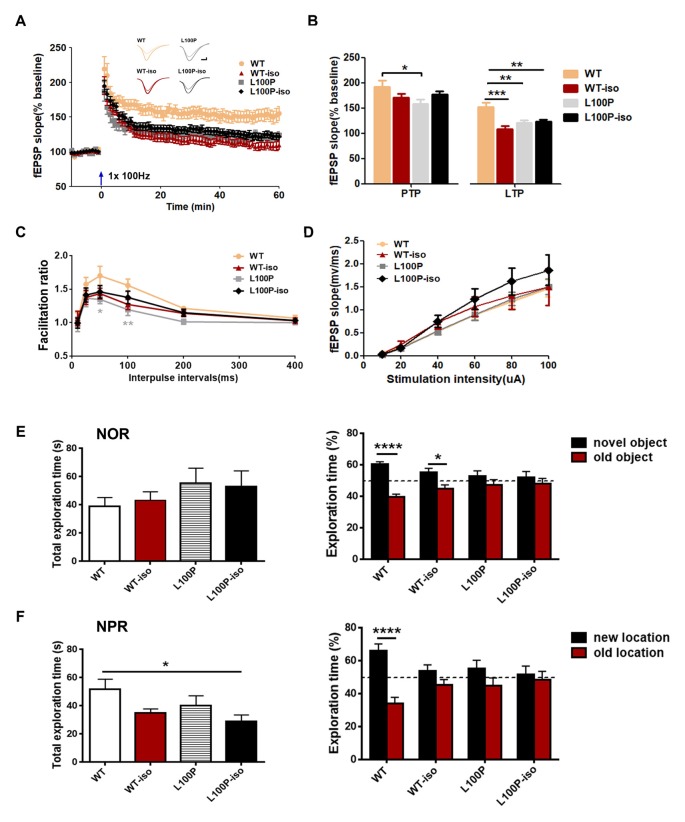
Adolescent isolation caused long lasting changes of synaptic plasticity in hippocampal SC-CA1 path. **(A)** LTP induced by 100 Hz tetanus. Insets, sample fEPSPs before, and 60 min after tetanus. **(B)** Post-tetanic potentiation (PTP; initial 5 min) and LTP (last 10 min). One-way ANOVA, *P < 0.05, **P < 0.01 or ***P < 0.001 compared to WT, *n* = 13, 13, 12, 17 slices from five mice per group. **(C)** PPR. Two-way ANOVA, *P < 0.05 at 50 ms interval and **P < 0.01 at 100 ms interval, *n* = 12, 13, 14, 16 slices from five mice per group. **(D)** input/output curves. Two-way ANOVA, *P* > 0.05, *n* = 27, 18, 20, 22 slices from six mice per group. **(E)** Novel object recognition (NOR) test. Left: total exploration time. One-way ANOVA, *P* > 0.05. Right: L100P and L100P-iso mice showed object recognition deficit, but not WT-iso mice. Two-way ANOVA, ****P < 0.0001 or *P < 0.05, *n* = 8 for each group. **(F)** Novel object-place recognition (NPR) test. Left: total exploration time. One-way ANOVA, *P < 0.05. Right: object-place recognition deficits in WT-iso, L100P and L100P-iso mice. Two-way ANOVA, ****P < 0.0001, *n* = 8 for each group. All data are shown as means ± SEM.

To test whether adolescent isolation affects synaptic plasticity and whether it works synergistically with L100P mutation to affect LTP, we measured fEPSPs in SC-CA1 synapses. LTP induced by 100 Hz stimulation was impaired, to the similar extent, in WT-iso, L100P and L100P-iso hippocampal slices (Figures 5A,B; one-way ANOVA for averaged last 10 min recordings after tetanus, *F*_(3,51)_ = 9.46, *P* < 0.0001; Dunnett’s Multiple Comparison Test, *q* = 3.58–5.13, *P* < 0.01–0.001 compared to WT). Statistical analysis showed that post-tetanic potentiation (PTP), a form of short-term enhancement mediated by certain presynaptic mechanism, was not altered in WT-iso and L100P-iso mice (Figure 5B; one-way ANOVA for averaged initial 5 min recording after tetanus, *P* > 0.05). Consistently, PPR measured in those two groups were also comparable to WT controls (Figure 5C; two-way ANOVA, *P* > 0.05), suggesting that the impaired LTP observed in WT-iso and L100P-iso mice were mainly due to post-synaptic mechanisms. Similar to our previous report, L100P mice showed significant suppression in both PTP (Figure 5B) and PPF (Figure 5C), suggesting that both pre- and post-synaptic mechanisms may underlie the LTP deficits of L100P mutants. We did not find significant changes in basal synaptic transmission since the synaptic input/output (I/O) curves was comparable among four groups (Figure 5D, two-way ANOVA, *F*_(3,498)_ = 2.43, *P* > 0.05). Therefore, our findings suggested that both adolescent isolation and L100P mutation cause long lasting changes of synaptic plasticity in hippocampal network, however the underlying mechanisms may be different.

### Adolescent Isolation Impaired NPR Memory in WT Mice

Since adolescent isolation triggered LTP deficits but it has no obvious effect on social memory of adult WT mice, we further checked whether it affects other hippocampus-dependent learning and memory processes. WT-iso mice exhibited normal performance in NOR test used to assess animals’ memory for familiar object (Figure 5E), but impairment in NPR test used to assess animals’ memory for object in familiar place (Figure 5F). Specifically, we found that while WT-iso mice spent more time exploring the novel object than the old one (Figure 5E; two-way ANOVA, *F*_(1,56)_ = 25.99, *P* < 0.0001; Sidak’s multiple comparisons test, *t* = 2.62, *P* < 0.05), it spent similar time exploring object at new location vs. at old location, suggesting object-place memory deficit (Figure 5F; two-way ANOVA, *F*_(1,56)_ = 19.70, *P* < 0.0001; Sidak’s multiple comparisons test, *t* = 1.65, *P* > 0.05). In contrast to WT mice, both L100P and L100P-iso mice showed profound deficits in NOR and NPR tests (Figures 5E,F), which is consistent with our previous finding. The total object exploration time of four groups were same in NOR test (Figure 5E, one-way ANOVA, *P* > 0.05), however the L100P-iso mice showed less exploration time in NPR tests (Figure 5F, one-way ANOVA, *F*_(3,28)_ = 2.949, *P* < 0.05; Dunnett’s multiple comparisons test, *q* = 2.86, *P* < 0.05 compared to WT). Therefore, our results indicated that adolescent isolation selectively impaired NPR memory while L100P mutation affects both NOR and NPR, the two tasks sharing many of the same motivational and visual-perceptual demands except that the latter is considered to be heavily dependent on hippocampus (Oliveira et al., 2010).

## Discussion

In this study, we reported for the first time that adolescent isolation interacts with *DISC1* L100P point mutation to specifically impair adult social memory, but has no effect on social interaction, anxiety- and depression-like behaviors. Further, we provided two lines of evidence demonstrating that certain genetic and psychosocial stressors interplays during adolescence to change neuronal networks activity, which may underlie the animal’s behavioral performance later. First, we found that adolescent isolation exacerbated the suppression of L100P point mutation on adult neurogenesis but had no effect on WT mice. Second, we found that adolescent isolation caused long-lasting changes of synaptic transmission specifically in hippocampal network of L100P mice, including reduced inhibitory synaptic transmission in both DG granule cells and CA1 pyramidal neurons, and reduced AMPA/NMDA ratio in CA1.

We injected BrdU at two different time windows with 3-weeks interval in order to differentiate BrdU^+^DCX^+^ and BrdU^+^NeuN^+^ cell population and be able to count both immature and mature newborn neurons at the same cohort of mice. To be noted, the total number and the percentage of BrdU^+^DCX^+^ cell may be underestimated in this study since we could not exclude the possibility that social isolation may affect the speed of newborn neuron maturation. In addition, we do not know how much those alterations in synaptic transmission could be attributed to impaired neurogenesis, however substantial evidence has suggested that adult-born granule cells, which is continuously generated from neural stem cells throughout life in all mammalians hippocampus including human, integrate in specific neuronal networks and participate in specific brain functions, including learning and memory (Harrison, 2004; Le Strat et al., 2009; Zhou et al., 2013). Therefore, we presume that improper adult neurogenesis caused by adolescent isolation may have a causal relationship with synaptic dysfunction in hippocampal network leading to social memory deficits in L100P mice. Importantly, physiological or pathological stimuli, such as stress, not only affect adult neurogenesis (Levone et al., 2015) but also neurogenesis in adolescence (Kozareva et al., 2018), presumably acting upon specific neuronal circuits and regulating distinct stage (Zhao et al., 2008; Ming and Song, 2011; Song and Rogulja, 2017).

Adolescence is generally believed to start on postnatal day 21 (P21). In particular, P21–P34 corresponds to early adolescence, P34–P46 to mid-adolescence and P46–P59 to late adolescence respectively (Burke et al., 2017). According to this criteria, the social isolation adopted in our study (from 5 weeks to 8 weeks of age) belongs to mid- to late-phase adolescent isolation, which is different from the majority of studies where isolation typically begins on the day of weaning (P21 or P28) and remains for 4 to 6 weeks or even longer (Burke et al., 2017). Previous studies in rats have reported that social isolation between weaning P21 to early adulthood P60 produces long-lasting changes on stress-related behaviors including anxiety and depression, which persist even after re-socialization (Burke et al., 2017), however in our study, we did not observe any change in anxiety- or depression-like behaviors in either L100P or WT mice undergoing social isolation from 5 weeks to 8 weeks of age, indicating that such mid- to late-phase adolescent isolation is rather mild in comparison to those highly stressful ones including maternal immune activation, long-term social isolation starting from weaning day, and chronic social defeat stress in adulthood, all of which can cause maladaptive structural and functional changes in the brain (Zhou et al., 2007; Haque et al., 2012; Lipina et al., 2013; Burke et al., 2017). Consistently, previous study reported that social isolation from 5 weeks to 8 weeks of age has no molecular, neurochemical and behavioral effect on WT mice (Niwa et al., 2013). Since strong environmental stressors cause cognitive and affective abnormality by itself (Haque et al., 2012; Lipina et al., 2013), which may confound the interplay between genetic and environmental risk factors on social brain and social behavior. Also, since we did not find social abnormality in homozygous L100P mice, we used mild social isolation stress and L100P homozygotes here. It is worth mentioning that in our study, after social isolation from 5 weeks to 8 weeks of age, mice remained to be single-housed for 4 weeks until being tested, meaning that the isolation lasts actually for 7 weeks. Such prolonged social isolation even starting at a late point of adolescence may be aversive enough to have substantial impact on synaptic transmission and plasticity in specific neuronal networks before obvious behavioral changes can be detected. In addition, it is not clear whether any observed effect can be attributed to isolation-induced disruption of particular development phases. Therefore, isolation during a sensitive period followed by a return to group housing before any evaluation may help to interpret related findings.

Previous studies have demonstrated that the hippocampus plays an essential role in social memory (Quiroga et al., 2005; Hitti and Siegelbaum, 2014; Okuyama et al., 2016; Piskorowski et al., 2016; Smith et al., 2016). Different from previous findings showing that prolonged early life social stress, for example maternal separation which mainly disrupts parenting, generally impairs LTP in the hippocampus while has no effect on synaptic transmission (Derks et al., 2017), we found that adolescent isolation which mainly disrupts conspecific social play, actually affects both synaptic transmission and plasticity. It seems that imbalanced synaptic transmission instead of LTP deficit in the hippocampal CA1 region is more related to social memory impairment in L100P-iso mice. Very recently, independent research groups reported that different sub-regions of the hippocampus play important while different roles in social memory process, in particular the dorsal hippocampus (Garrido Zinn et al., 2016), ventral CA1 (Okuyama et al., 2016) and CA2 (Hitti and Siegelbaum, 2014; Piskorowski et al., 2016; Smith et al., 2016; Leroy et al., 2017). Therefore, it is interesting to work on different hippocampal sub-regions and check synaptic function changes specifically in those microcircuits.

So far, how gene-environment interaction leads to social memory deficit is still unknown. Accumulating evidence indicates that DISC1, an intracellular scaffold protein, acts in concert with numerous interacting proteins to regulate neurogenesis, neuronal migration and neurite outgrowth in developmental brain, and synaptic function in adulthood (Kim et al., 2009; Brandon and Sawa, 2011; Ishizuka et al., 2011). Meanwhile, social experience during adolescence alters stress-related neural circuits and monoaminergic systems (Burke et al., 2017). We presumed that dysregulation of synaptic function in the hippocampus triggered by *DISC1* mutation × adolescence isolation contributes to social memory deficit observed in adult mice. In addition, stress-induced epigenetic modulation of dopaminergic neurons in the ventral tegmental area may also play certain role in this process (Niwa et al., 2013).

In summary, our study indicates that social isolation during late adolescence elicits social memory deficits in adult when combined with an appropriate genetic risk, for example, *DISC1* L100P point mutation. The interaction between those two factors damages neurogenesis, synaptic transmission and plasticity in hippocampal networks, which may underlie social memory deficits in adults. Nevertheless, further experiments are required to clarify the causal relationship between such behavioral deficits and abnormal adult neurogenesis, synaptic transmission and plasticity observed in the hippocampus.

## Author Contributions

NL, LG and HG performed all the electrophysiological experiments. GS and LC performed all the behavioral experiments, immunostaining and imaging study. NL and HC performed all the data analyses. YZ and G-DL supervised the experiments and wrote the manuscript.

## Conflict of Interest Statement

The authors declare that the research was conducted in the absence of any commercial or financial relationships that could be construed as a potential conflict of interest.

## Footnotes

^1^https://www.brc.riken.jp/lab/animal/en/
